# Whole Transcriptome Mapping Identifies an Immune- and Metabolism-Related Non-coding RNA Landscape Remodeled by Mechanical Stress in IL-1β-Induced Rat OA-like Chondrocytes

**DOI:** 10.3389/fgene.2022.821508

**Published:** 2022-03-03

**Authors:** Jiaming Zhang, Xiaoxia Hao, Ruimin Chi, Jiawei Liu, Xingru Shang, Xiaofeng Deng, Jun Qi, Tao Xu

**Affiliations:** ^1^ Department of Orthopedics, Tongji Hospital, Tongji Medical College, Huazhong University of Science and Technology, Wuhan, China; ^2^ Cancer Center, Union Hospital, Tongji Medical College, Huazhong University of Science and Technology, Wuhan, China; ^3^ Department of Rehabilitation, Tongji Hospital, Tongji Medical College, Huazhong University of Science and Technology, Wuhan, China

**Keywords:** osteoarthritis, chondrocyte biology, mechanical stress, transcriptome, non-coding RNA

## Abstract

**Background:** Osteoarthritis (OA) is a common degenerative joint disease. The aims of this study are to explore the effects of mechanical stress on whole transcriptome landscape and to identify a non-coding transcriptome signature of mechanical stress.

**Methods:** Next-generation RNA sequencing (RNA-seq) was performed on IL-1β-induced OA-like chondrocytes stimulated by mechanical stress. Integrated bioinformatics analysis was performed and further verified by experimental validations.

**Results:** A total of 5,022 differentially expressed mRNAs (DEMs), 88 differentially expressed miRNAs (DEMIs), 1,259 differentially expressed lncRNAs (DELs), and 393 differentially expressed circRNAs (DECs) were identified as the transcriptome response to mechanical stress. The functional annotation of the DEMs revealed the effects of mechanical stress on chondrocyte biology, ranging from cell fate, metabolism, and motility to endocrine, immune response, and signaling transduction. Among the DELs, ∼92.6% were identified as the novel lncRNAs. According to the co-expressing DEMs potentially regulated by the responsive DELs, we found that these DELs were involved in the modification of immune and metabolism. Moreover, immune- and metabolism-relevant DELs exhibited a notable involvement in the competing endogenous RNA (ceRNA) regulation networks. Silencing lncRNA *TCONS_00029778* attenuated cellular senescence induced by mechanical stress. Moreover, the expression of *Cd80* was elevated by mechanical stress, which was rescued by silencing *TCONS_00029778*.

**Conclusion:** The transcriptome landscape of IL-1β-induced OA-like chondrocytes was remarkably remodeled by mechanical stress. This study identified an immune- and metabolism-related ncRNA transcriptome signature responsive to mechanical stress and provides an insight of ncRNAs into chondrocyte biology and OA.

## Introduction

Osteoarthritis (OA) is a common degenerative joint disease characterized by degradation of articular cartilage and subchondral bone, which causes a significant socioeconomic burden (Felson, 2010; Glyn-Jones et al., 2015). Mechanical stimulation from daily activities and physical exercise is indispensable for articular cartilage fitness. Physical exercise also gives promise to OA management by alleviating symptoms and structural progress (Ramage et al., 2009; Iijima et al., 2016; Chang et al., 2017). However, excessive mechanical loading is a critical risk factor of OA resulting in the damaged integrity of cartilage-subchondral bone unit, characterized by chondrocyte death, cartilage degeneration, and subchondral bone remodeling (Sharma et al., 2013; Poulet et al., 2015; Jorgensen et al., 2017).

As a key component of cartilage, chondrocytes are mechanosensitive, perceiving and responding to mechanical stress throughout life in a magnitude-dependent manner (Liu et al., 2016b). It is still a puzzle how the mechanical stimulation favors the cartilage and chondrocytes and how this process is attenuated and the outcome is turned over by excessive loading. In principle, moderate mechanical stress favors chondrocytes by regulating multiple signaling pathways and cellular functions, such as cytoskeleton and primary cilia (Yang et al., 2016; Xiang et al., 2018; Fu et al., 2019), while excessive mechanical stimulation always triggers the unfavorable changes and outcomes of chondrocytes, for instance, enhanced catabolic effects, compromised respiratory function, and apoptosis (Sun, 2010; Coleman et al., 2016). Mitochondrial dysfunction is a well-accepted hallmark of OA (Buckwalter et al., 2013). Mitochondrial fitness is critical for chondrocyte fate and functions, at least, by regulating metabolism (Zhang et al., 2021). Under unfavorable stress, chondrocytes not only increase reactive oxygen species (ROS) production, but also undergo metabolism shift from oxidative phosphorylation to glycolysis *via* the AMP-activated protein kinase (AMPK) and mechanistic target of rapamycin (mTOR) pathways (Zheng et al., 2021). Moreover, mitochondrial mechanotransduction is also critical for chondrocyte biology in the pathogenesis of OA. Activating by ROS/Liver kinase B1 (LKB1) and Ca^2+^/calcium/calmodulin-dependent kinase kinase 2 (CAMKK2), AMPK signaling pathway and subsequent ECM production, cytoskeleton rearrangement, and mitochondrial fitness. AMPK signaling pathway may be at the core of the events in which mechanical stimulation elicits alterations in mitochondrial functions, while the mechanisms of reduced AMPK activity in OA are elusive (Jiang et al., 2021).

Due to the application of high-throughput methods, increasing numbers of disturbed signaling pathways and mechanosensitive genes are highlighted, including long-noncoding RNAs (lncRNA). lncRNAs are broadly defined as a class of non-coding transcripts more than 200 nucleotides in length without coding potentiality (Zhang J et al., 2018). Liu et al. (2016a) identified that 107 lncRNAs were differentially expressed in the damaged cartilage and increased lncRNA MRS was responsive to cyclic tensile strain and involved in extracellular matrix (ECM) degradation by disrupting cytoskeleton and increasing matrix metalloproteinases (MMPs). Qiu et al. (2021) identified 16 upregulated and two downregulated miRNAs, as well as 44 upregulated lncRNAs and 39 downregulated lncRNAs in rat femur and tibia under mechanical stress. These lines of evidence suggest the underlying involvement of lncRNAs in mechanotransduction. However, the comprehensive understanding of mechanical response of chondrocyte transcriptome landscape, including mRNAs, microRNAs (miRNAs), lncRNAs, and circular RNAs (circRNAs), is still absent. The roles of noncoding RNAs (ncRNAs) in mechanotransduction remain elusive, while some ncRNAs were identified as the indispensable participators in OA pathogenesis (Li et al., 2018; Malemud, 2018; Kong et al., 2021).

In this study, we employed next-generation sequencing (RNA-seq) to explore the effects of mechanical stress on whole transcriptome landscape and to identify a non-coding transcriptome signature of mechanics response. This study identified the immune- and metabolism-related lncRNAs that may be involved in the mechanisms upon overloading and provides an insight of ncRNAs into chondrocyte biology and OA.

## Materials and Methods

### Isolation and Culture of Chondrocytes

Chondrocytes were isolated from the knee articular cartilage of 3-day-old Sprague–Dawley rats. The animal protocol was approved by the Experimental Animal Ethics Committee of Tongji Medical College, Huazhong University of Science and Technology (Wuhan, China). Briefly, cartilage tissue was minced and digested with 0.25% trypsin (Gibco, United States) for 30 min and 0.25% collagenase II (Invitrogen, United States) at 37°C for 6 h sequentially. Chondrocytes were collected and cultured in an incubator containing 5% CO_2_ at 37°C with Dulbecco’s DMEM/F12 (HyClone, United States) supplemented with10% fetal bovine serum (FBS, Gibco, United States) and antibiotics (100 U/ml penicillin G sodium, 100 μg/ml streptomycin sulfate) (Gibco, United States).

### IL-1β Inducement on Rat Chondrocyte

It is well-documented that IL-1β can induce OA-like phenotypes in cultured chondrocytes, characterized by the reduction of anabolism (decreased ECM components) and the increase of catabolism (increased metalloproteinases) (Yao et al., 2019; Yao et al., 2021; Zhang et al., 2021). The concentrations of IL-1β ranging from 1 ng/ml to as much as 1,000 ng/ml, compared to <10 pg/ml in body fluids, are considered as supra-physiologic concentrations. Therefore, supra-physiologic concentrations of IL-1β (5–10 ng/ml) are required to induce OA-like changes in cartilage explants and chondrocyte cultures. In our study, chondrocytes were induced by recombinant rat IL-1β (5 ng/ml) (Catalog No. 400-01B, Peprotech, United States) for 24 h to obtain OA-like phenotypes (Yao et al., 2019).

### Mechanical Stress Stimulation

Mechanical stress was applied to IL-1β-induced chondrocytes by using the four-point bending system as our previous studies described (Xu et al., 2013). Briefly, 1×10^6^ chondrocytes at passage first were placed on a special vinyl plate (7.5 cm × 4 cm) and cultured until reaching 80% confluence. Then, the vinyl plate was transferred to a bending dish filled with 20 ml of DMEM/F12 medium in an incubator containing 5% CO_2_ at 37°C. Chondrocytes were exposed to mechanical stress with intensity of 2,000 μstrain at a frequency of 1 Hz for 4 h. For the control group, cells were cultured in the same condition without mechanical stress stimulation for 4 h.

### RNA Isolation and Real-Time Polymerase Chain Reaction (RT-PCR)

Total RNA was extracted immediately after IL-1β intervention (24 h) and mechanical stress stimulation (4 h). Total RNAs were extracted using RNeasy kit (Qiagen, Germany) according to the manufacturer’s instructions. Reverse transcription (RT) was performed using QuantiTect Reverse Transcription kit (Qiagen, Germany). RT-PCR was performed using CFX Connect Real-Time System (Bio-Rad, Singapore) with SYBR green master mix (TOYOBO, Japan) as previously described (Yao et al., 2019). The thermocycling conditions were as follows: 30 s of polymerase activation at 95°C, followed by 40 cycles of 95°C for 5 s and 60°C for 30 s. Gene expression was normalized to *Gapdh* and calculated according to the 2^−ΔΔCT^ method. The primers used in this study are listed in Supplementary Table S1. Each experiment was repeated three times.

### Small Interfering RNA Silencing

The small interfering RNA (siRNA) used in this study was purchased from Ribbio Biological (Guangzhou, China). siRNA-TCONS_00029778: GCA​CCA​GAA​ATG​CCA​TGA​A. Transfection was preformed using Lipofectamine 3000 transfection reagent according to manufacturer’s protocol (Guo et al., 2020). Forty-eight hours after transfection, the cells were subjected to IL-1β inducement and mechanical stimulation.

### β-Galactosidase Staining

β-Galactosidase staining was performed to assess cellular senescence of the mechanical stress-stimulated chondrocytes. The staining was performed according to the manufacturer’s instructions (Senescence β-Galactosidase Staining Kit #9860, Cell Signaling, United States) (Itahana et al., 2007). The average number of senescent chondrocytes quantified in four independent fields selected by a blind way was used to represent one individual experiment. Five individual experiments were repeated.

### Mitochondrion and Lysosome Staining

The subcellular location and morphology of mitochondrion and lysosome was accessed by Mito-Tracker Green (Beyotime, China) and Lyso-Tracker Red (Beyotime, China) staining according to the manufacturer’s instructions (Lin et al., 2021; Zhang et al., 2021). Lysosome number of 50 chondrocytes were quantified in a double-blind way by two individual researchers. The mean values were calculated by the data of 50 cells in an individual experiment. Five individual experiments were repeated.

### Next Generation RNA-Sequencing (RNA-Seq)

Total RNA was isolated by Trizol (Invitrogen, United States) according to the manufacturer’s instructions. Each group contains three biological replicates. Library construction followed the standard protocols (Djebali et al., 2012) and was sequenced on the Illumina HiSeq 4000 platform by Genedenovo Biotechnology Co., Ltd (Guangzhou, China). We used fastp tool (v0.18.0) (Chen et al., 2018) to filter for high-quality reads by trimming adapters and removing reads that contained >10% unknown nucleotides, and reads with >50% low quality (*Q*-value ≤ 20) bases. Clean paired-end reads were then mapped to the *Rattus norvegicus* reference genome (Ensembl release 91 Rnor 6.0) with HISAT2 v.2.1.0 (Kim et al., 2015) with default parameters. The resulting files were sorted using SAMtools (v1.1.1) and subjected to HTSeq (0.9.0) to obtain the counts of each gene.

Raw count was filtered by a minimum expression threshold of 5 for coding genes and 2 for non-coding genes. The genes with low count (<5 or <2) in both groups were removed. The principal component analysis (PCA) was performed to determine the overall difference between the mechanical stress-stimulated group and control (David and Jacobs, 2014). Differential expression analysis of gene abundances was performed by R package *edgeR* (v3.11) (Nikolayeva and Robinson, 2014). Differentially expressed transcripts were defined by the criteria of |log_2_ (fold change)| > 1 (|log_2_ FC| > 1) and FDR < 0.05 for mRNA and lncRNA, *p*-value < 0.05 for miRNA and circRNA, respectively. The expression of transcripts was visualized by the R package *pheatmap* (v1.0.12) (Li et al., 2020). Gene Ontology (GO) and the Kyoto Encyclopedia of Genes and Genomes (KEGG) enrichment analyses were performed by the R package *clusterProfiler* (v3.11) (Yu et al., 2012). Also, Gene Set Enrichment Analysis (GSEA) was employed to determine whether an *a priori* defined set of genes shows statistically significant differences between the groups or samples by GSEA software (v4.1.0) (Subramanian et al., 2005).

### Novel lncRNA Identification

Th software Coding-Non-Coding Index (CNCI) (v2) (Sun et al., 2013) and Coding Potential Calculator (CPC) (Kong et al., 2007) were used to assess the protein-coding potential of novel transcripts by default parameters. Meanwhile, novel transcripts were mapped to SwissProt (http://www.expasy.ch/sprot/) sequence database to provide a high level of protein annotation (Bairoch and Apweiler, 2000). The intersection of both non-protein-coding potential results and non-protein annotation results were defined as the novel lncRNAs.

### Regulation Mechanism Analysis and Function Annotation of lncRNAs

Antisense lncRNAs potentially regulate the host gene expression by silencing transcription and mRNA stability. In order to reveal the interaction between antisense lncRNA and mRNA, the software RNAplex (v2.4.16) was used to predict the complementary correlation of antisense lncRNA and mRNA based on the calculation of minimum free energy through thermodynamics structure (Tafer and Hofacker, 2008). Also, lncRNAs regulate their neighboring genes on the same allele in a *cis-*regulation mechanism. lncRNAs in less than 100 kb up-/downstream of a gene are likely to be *cis*-regulators. Moreover, lncRNAs regulate the expression of non-adjacent genes in a *trans-*regulation mechanism (Kopp and Mendell, 2018). The protein-coding genes that are potentially regulated by lncRNAs in *antisense*, *cis-*, or *trans-*mechanisms and also co-expression with corresponding lncRNAs were defined as the target genes of lncRNAs. The correlation between lncRNA and mRNA was calculated using the Pearson correlation coefficient and *p*-value. The Pearson correlation value was >0.9 or <−0.9, and *p*-value < 0.05 indicated that there could be a significant correlation between lncRNA and mRNA. The target genes of lncRNAs were subjected to enrichment analysis of GO functions and KEGG pathways.

### CeRNA Network Mining and Function Annotation

Before ceRNA network construction, the targets of DEMIs were predicted by RNAhybrid (v2.1.2)/svm_light (v6.01), Miranda (v3.3a), and TargetScan (v7.0). Ritchie (2017) define the miRNA-mRNA/lncRNA/circRNA pairs based on complementary bases. The DEMs potentially regulated by DECs were defined based on expression correlation evaluated by Pearson correlation coefficient.

The principles of ceRNA network construction were strong binding potential and expression relevance. Expression correlation between DEMIs and DEMs/DELs/DECs was evaluated by the Pearson correlation coefficient (*R*). Pairs with *R* < −0.9 were selected as co-expressed miRNA-mRNA/lncRNA/circRNA pairs, among which the pairs with positively co-expressed mRNA-lncRNA/circRNA were defined as ceRNA pairs. To increase the accuracy of prediction, hypergeometric cumulative distribution function test was performed as previous study described (Xu et al., 2016). *p*-value less than 0.05 was considered significant. To functionally annotate the ceRNA network, GO enrichment and KEGG pathway analysis of the DEMs in the ceRNA network were performed. Transcript connectivity in the ceRNA network was defined as number of co-expressed targeted miRNAs, which was employed to assess the significance of genes in biological networks. The ceRNA network was visualized by the R package Circlize (Version 0.4.13) (Gu et al., 2014).

### Statistical Analysis

The statistics methods used in this study include unpaired two-tailed Student’s *t*-test and one-way ANOVA with Dunnett’s multiple comparisons test. *p* < 0.05 was considered statistically significant. The application of each statistics method was specified in figure legends. GraphPad Prism 7.0 was used for the statistical analysis, and data were presented as means ± SD.

## Results

### Hallmark Remodeling of RNA Whole Transcriptome Landscape in IL-1β-Induced Rat OA-like Chondrocytes as a Response to Mechanical Stress

**FIGURE 1:**
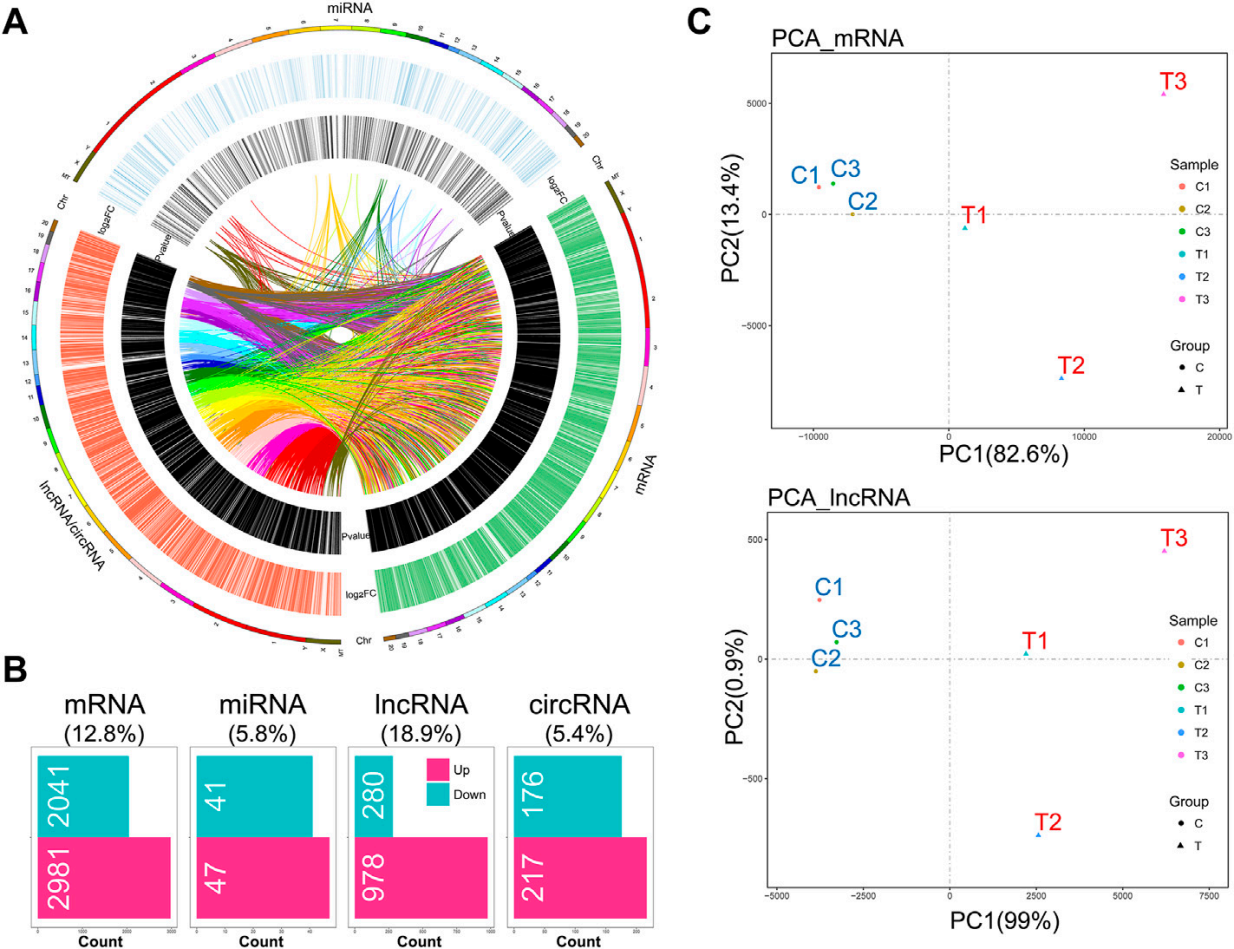
Hallmark remodeling of RNA whole transcriptomic landscape in OA-like chondrocytes as a response to mechanical stress. **(A)** Transcriptomic landscape of mechanical stress-stimulated OA-like chondrocytes, including mRNA, miRNA, and lncRNA/circRNA, based on gene co-expression analysis. **(B)** A total of 5,022 differentially expressed mRNAs (DEMs), 88 differentially expressed miRNAs (DEMIs), 1,259 differentially expressed lncRNAs (DELs), and 393 differentially expressed circRNAs (DECs) were identified in the comparison of the mechanical stress-stimulated group and the control group (FDR < 0.05, |log2FC| > 1). **(C)** Principal component analysis of the mechanical stress-stimulated group and the control group based on mRNA profiles or lncRNA profiles. The mRNA transcriptome shows high relevance with the lncRNA transcriptome of OA-like chondrocytes.

Given that the mechanical stimulation employed in this study induced the genes relevant to OA-like phenotypes, including *Mmp9* and *Mmp13* (encoding MMP9 and MMP13), we found that this stimulation promoted chondrocyte catabolic metabolism (Supplementary Figure S1A). Also, senescent chondrocytes were increased under this stimulation (Supplementary Figure S1B). RNA-seq analysis showed a distinct whole transcriptome profile of mechanical stress-stimulated OA-like chondrocytes, which included a total of 5,022 differentially expressed mRNAs (DEMs), 88 differentially expressed miRNAs (DEMIs), 1,258 differentially expressed lncRNAs (DELs), and 393 differentially expressed circRNAs (DECs) (Supplementary Table S2), and the extensive crosstalk among these four compositions by co-expressing relation (Figures 1A,B). Principal component analysis based on mRNA or lncRNA profiles showed the high relevance of lncRNA transcriptome with mRNA in IL-1β-induced rat OA-like chondrocytes (Figure 1C).

### The mRNA Transcriptome Reveals the Critical Mechanical Stress-Responsive Genes and Signaling Pathways

**FIGURE 2:**
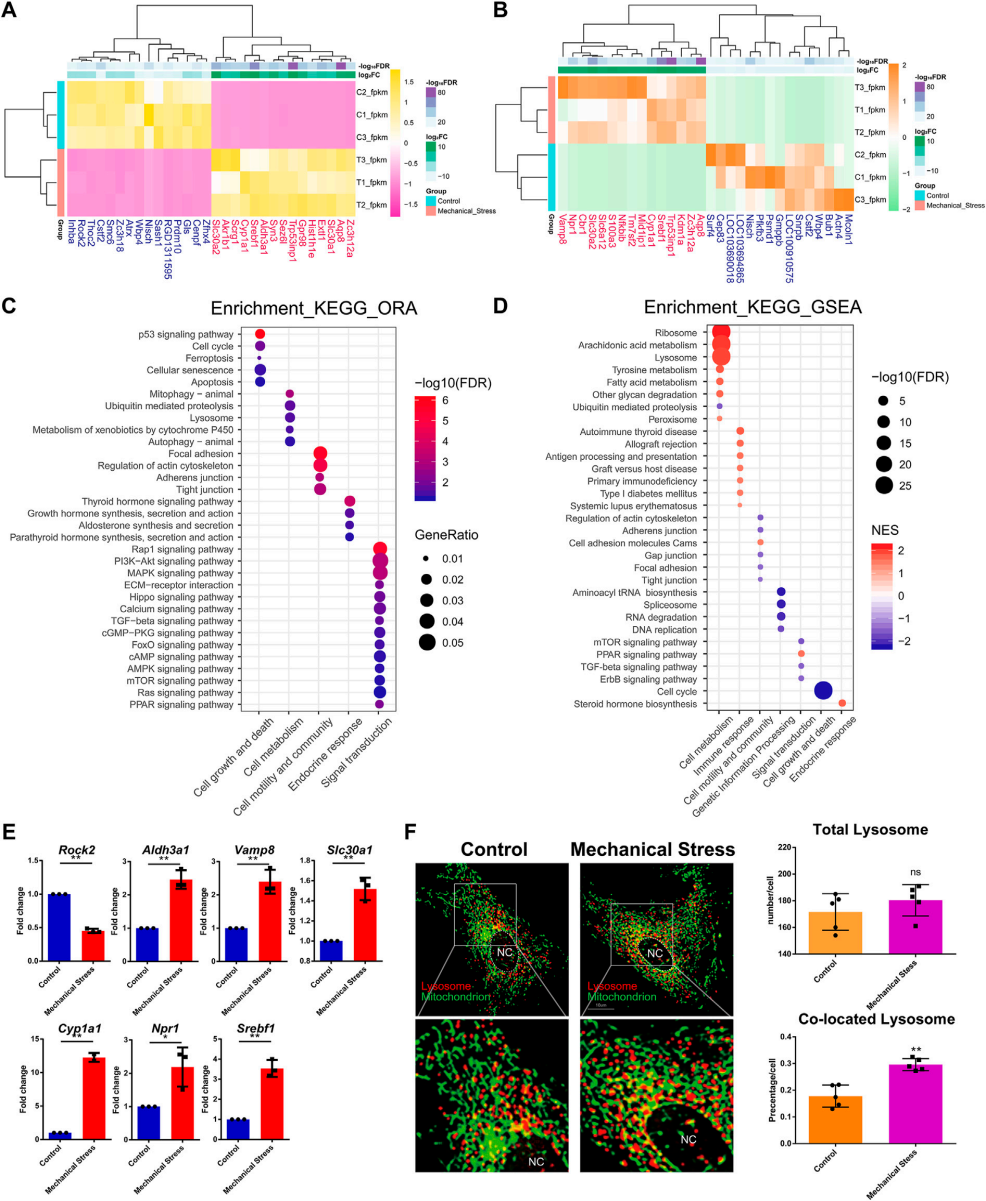
The mRNA transcriptome reveals the critical mechanical stress-responsive genes and signaling pathways. **(A)** Top 15 up- or downregulated genes in the rank of FDR. **(B)** Top 15 up- or downregulated genes in the rank of log2FC. **(C)** KEGG pathway analysis of DEMs by ORA method. The relevant pathways are assigned into five groups, namely, cell growth and death, cell metabolism, cell motility and community, endocrine response, and signaling transduction. The color and size of dot represent FDR and GeneRatio of the corresponding KEGG pathway, respectively. FDR, false discovery rate, *p*-value estimated by one-way ANOVA and adjusted by the Benjamini–Hochberg method. GeneRatio indicates the gene number ratio in each KEGG pathway. **(D)** KEGG pathway analysis of total gene expression profile by the GSEA method. The relevant pathways are assigned into seven groups, namely, cell growth and death, cell metabolism, cell motility and community, genetic information processing, endocrine response, immune response, and signaling transduction. The color and size of dot represent NES and FDR of the corresponding KEGG pathway, respectively. NES, normalized enrichment score. A positive NES indicates that genes over-represented in the gene set are mostly upregulated in the comparison of mechanical stress versus control and a negative NES instead indicated the opposite. FDR, false discovery rate, *p*-value estimated by one-way ANOVA and adjusted by the Benjamini–Hochberg method. **(E)** RT-PCR validation of the essential genes contributing to relevant signaling pathway responsive to mechanical stress, including *Rock2*, *Aldh3a1*, *Vamp8*, *Slc30a1*, *Cyp1a1*, *Npr1*, and *Srebf1*. ^*^ *p* <0.05, ^**^ *p* <0.01, two-tailed Student’s *t*-test, mechanical stress versus control. **(F)** Lysosome (red) and mitochondrion (green) staining with or without mechanical stimulation. Total number of lysosomes and the colocation events with mitochondria is quantified in each chondrocyte. ns, no significance; ^**^ *p* <0.01, two-tailed Student’s *t*-test; mechanical stress versus control. nc, nucleus.

To understand the response of IL-1β-induced rat OA-like chondrocytes to mechanical stress, we first focused on the mRNA transcriptome and defined the top 15 up- or downregulated genes in the rank of FDR or log_2_FC as the outstanding responsive genes (Figures 2A,B), among which *Srebf1* (Sterol regulatory element-binding transcription factor 1), *Trp53inp1* (Tumor protein P53 inducible nuclear protein 1), *Slc30a2* (Solute carrier family 30 member 2), *Cyp1a1* (Cytochrome P450 family subfamily A member 1), *Zc3h12a* (Zinc finger CCCH-type containing 12A), *Aqp8* (Aquaporin 8), *Nisch* (Nischarin), *Cstf2* (Cleavage stimulation factor subunit 2), and *Wbp4* (WW domain binding protein 4) were identified as the significant genes based on the rank of FDR and FC (Table 1, Supplementary Figure S2).

**TABLE 1:** Notable differentially expressed coding genes responsive to mechanical stress.

Gene symbol	log_2_FC	FDR
*Srebf1*	14.47	9.04E-66
*Trp53inp1*	14.36	9.09E-83
*Slc30a2*	14.29	7.49E-49
*Cyp1a1*	13.3	1.22E-34
*Zc3h12a*	12.67	6.02E-29
*Aqp8*	12.29	1.51E-86
*Nisch*	−12.5	6.23E-28
*Cstf2*	−12.26	5.84E-32
*Wbp4*	−11.66	1.13E-16

FC, the fold change of mechanical stress compared to control. FDR, *p*-value adjusted by the Benjamini–Hochberg method. FDR < 0.05 was considered statistically significant.

GO analysis of DEMs showed that mechanical stimulation is highly relevant to the biological process, such as response to stress, cell death, and biosynthetic and metabolic process (Supplementary Figure S3). Moreover, mechanical stimulation regulated enzyme binding, kinase activity, GTPase activity, and oxidoreductase activity (Supplementary Figure S3), suggesting a connection between mechanical stress and cellular metabolism. KEGG pathway analysis by the ORA method showed that the mechanical stress-relevant pathways were assigned into five categories, including cell growth and death, cell metabolism, cell motility and community, endocrine response, and signaling transduction, among which p53 signaling pathway, mitophagy, focal adhesion, thyroid hormone signaling pathway, and Rap1 signaling pathway were the most significant pathway in each category, respectively (Figure 2C). Given that a large amount of DEMs were involved in PI3K-Akt (357 DEMs, not shown) and MAPK signaling pathway (304 DEMs, not shown), these two pathways were considered as widely disturbed by mechanical stress.

Given that the ORA method only considers a group of pre-selected genes by a specific criterion (FDR < 0.05 and log_2_FC < −1 or >1), we also employed the GSEA method to explore the pathways relevant to global change of transcriptome. Different with the pathway categories identified by the ORA method, which were considered as the robust response of signaling pathway, three more categories, including genetic information processing, endocrine, and immune response, were further identified (Figure 2D; Supplementary Figure S4,S5). From the category perspective, cell metabolism, immune, and endocrine response were almost enhanced, while cell growth and death, cell motility and community, genetic information processing, and signaling transduction were almost suppressed. Specifically, the functions of ribosome and lysosome and the cellular metabolism, such as arachidonic acid, tyrosine, and fatty acid metabolism, were potentially activated, while ubiquitin-mediated proteolysis was assumed to be inhibited. Also, cell cycle and relevant pathways, such as DNA replication, were suppressed by mechanical stress, which is consistent with the item “cellular senescence” in the ORA results. Regarding the signaling pathways, mTOR, TGF-beta, and ErbB signaling pathways were likely to be suppressed, while PPAR signaling pathway was potentially activated.

The ten genes relevant to the signaling pathways responsive to mechanical stress (Figures 1A, 2B), namely, *Gls*, *Mcoln1*, *Actn4*, *Rock2*, *Aldh3a1*, *Vamp8*, *Slc30a1*, *Cyp1a1*, *Npr1*, and *Srebf1*, were selected for further verification by RT-PCR. Besides *Gls*, *Mcoln1*, and *Actn4*, seven out of ten were validated. There was increased expression of *Aldh3a1*, *Vamp8*, *Slc30a1*, *Cyp1a1*, *Npr1*, and Srebf1 and downregulation of *Rock2* under mechanical stress (Figure 2E). Moreover, we found that the lysosomes co-located with mitochondria were increased while there was no difference in the total number of lysosomes between the stimulated and the control groups (Figure 2F).

### Novel lncRNAs Emerged as a Marked Response to Mechanical Stress

**FIGURE 3:**
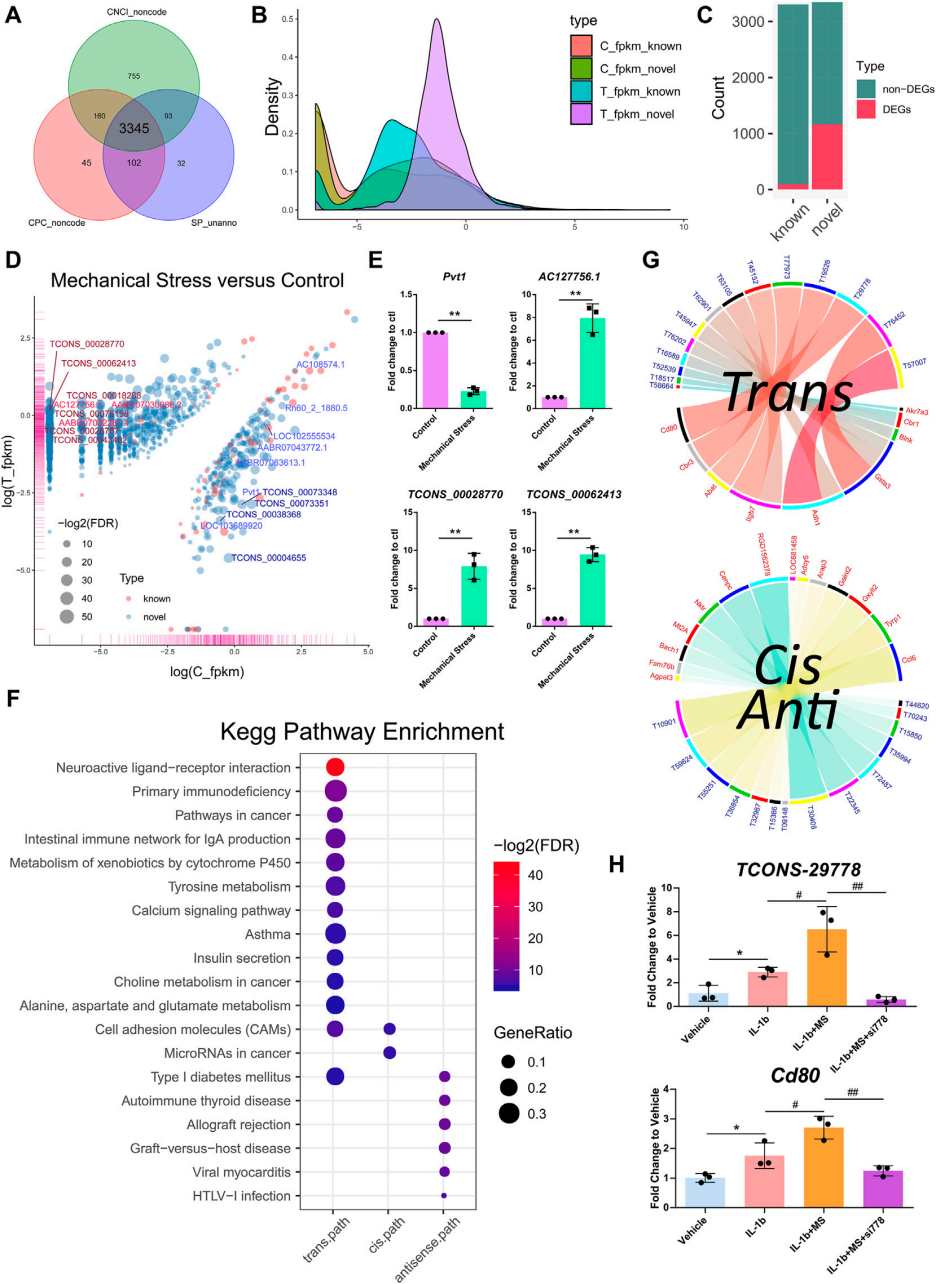
Novel lncRNAs emerged as a key response to mechanical stress. **(A)** A total of 3,345 novel lncRNAs are identified by software CNCI, CPC, and the SwissProt database. **(B)** The density plot of the gene expression of the known and the novel lncRNAs. Mechanical stress increases the relatively high expressed lncRNAs and decreases the relatively low expressed lncRNAs, including the known and the novel lncRNAs. The novel lncRNAs are the most significantly altered composition responsive to mechanical stress. **(C)** The numbers of the non-DELs and DELs in the known and the novel groups. The number of DELs in the novel group is notably higher than the known group. **(D)** The volcano plot of DELs with FDR < 0.05 and log_2_FC > 1 or < −1 in the comparison of mechanical stress versus control. The red bubble represents the known lncRNA. The blue bubble represents the novel lncRNA. The upregulated known and novel lncRNAs are labeled by bright red and dark red, respectively. The downregulated known and novel lncRNAs are labeled by bright blue and dark blue, respectively. **(E)** RT-PCR validation of the known lncRNA *Pvt1* and *AC127756.1* and the novel lncRNA *TCONS_00028770* and *TCONS_00062413*. ^*^ *p* <0.05, ^**^ *p* <0.01, two-tailed Student’s *t*-test, mechanical stress versus control. **(F)** The KEGG pathway analysis of the coding genes potentially regulated by mechanical stress-responsive lncRNAs in *trans-*, *cis-*, and *antisense* manners by the ORA method. The color and size of dot represent FDR and GeneRatio of the corresponding KEGG pathway, respectively. FDR, false discovery rate, *p*-value estimated by one-way ANOVA and adjusted by the Benjamini–Hochberg method. GeneRatio indicates the gene number ratio in each KEGG pathway. **(G)** Co-expression of lncRNA–mRNA pairs in *trans-*, *cis*-, or *antisense* regulation. Blue and yellow bands represent the *cis-* and *antisense* pairs, respectively. Red and blue label represent mRNA and lncRNA, respectively. T58664, the abbreviation of lncRNA *TCONS_00058664*. **(H)** RT-PCR validation of the lncRNA–mRNA *TCONS_00029778*-*Cd80*. MS, mechanical stress. ^*^ *p* <0.05, two-tailed Student’s *t*-test; ^#^ *p* <0.05, ^##^ *p* <0.01, one-way ANOVA.

Among the 6,649 lncRNA transcripts we detected, a total of 3,345 novel lncRNAs were identified by software CNCI, CPC, and the SwissProt database, accounting for more than 50% in total (Figure 3A). The density plot of the gene expression of these lncRNAs showed that mechanical stress increased the relatively high expressed lncRNAs and decreased the relatively low expressed lncRNAs, including both the known and the novel lncRNAs. The novel lncRNAs were the most significantly altered composition responsive to mechanical stress (Figure 3B). Moreover, as shown in Figure 3C, the number of novel DELs in the novel group was notably higher than the known lncRNAs. In this context, we considered that the novel lncRNAs may be the key response to mechanical stress. As shown in Figure 3D, the volcano plot of DELs showed the top 10 up- or downregulated known and novel DELs ranked by FDR, respectively. The top 10 known or novel DELs are summarized in Table 2. Among them, the known lncRNA *AC127756.1* (top 1) and *Pvt1* (top 3) and the novel lncRNA *TCONS_00028770* (top 1) and *TCONS_00062413* (top 2) were further validated by RT-PCR (Figure 3E). To functionally annotate these DELs, the coding genes that are potentially regulated by mechanical stress-responsive lncRNAs in *trans-*, *cis-*, and *antisense* manners were identified and further involved in KEGG pathway analysis. As shown in Figure 3F and Supplementary Figure S6, DELs were highly involved immune-relevant pathways, such as primary immunodeficiency, intestinal immune network for IgA production, autoimmune thyroid disease, allograft rejection, and graft-versus-host disease, and metabolism pathways, such as metabolism of xenobiotics by cytochrome P450 and tyrosine metabolism. Also, the DEM–DEL pairs involved in these immune and metabolism pathways were plotted in Figures 3E,G. *TCONS_00016589*-*Blnk*, *TCONS_00077973*-*Itgb7*, *TCONS_00063105*-*Itgb7*, *TCONS_00029778*-*Cd80*, *TCONS_00015850*-*Bach1*, *TCONS_00035994*-*Mt2A*, *TCONS_00072487*-*Nktr*, *TCONS_00032987*-*Arap3*, *TCONS_00036854-Galnt2*, and *TCONS_00010901-Ccl6* were identified as the immune-relevant lncRNA-coding gene pairs according to the correlation. Interestingly, silencing lncRNA *TCONS_00029778* attenuated cellular senescence induced by mechanical stress (Supplementary Figure S7). Moreover, the expression of *Cd80* was elevated by mechanical stress, which was recused by silencing lncRNA *TCONS_00029778* (Figure 3H).

**TABLE 2:** Notable differentially expressed long non-coding RNA responsive to mechanical stress.

Gene symbol	log_2_FC	FDR	Category
*AC127756.1*	10.05	2.22E-08	Known
*LOC103689920*	−4.87	2.53E-08	Known
*Pvt1*	−5.20	7.80E-08	Known
*Rn60_2_1880.5*	−2.57	3.23E-07	Known
*AABR07063613.1*	−2.42	3.73E-07	Known
*AABR07032261.4*	6.96	3.73E-07	Known
*AABR07030086.2*	4.89	4.42E-07	Known
*AC108574.1*	−1.97	1.41E-06	Known
*LOC102555534*	−2.39	1.84E-06	Known
*AABR07043772.1*	2.35	4.02E-06	Known
*TCONS_00028770*	10.20	2.83E-18	Novel
*TCONS_00062413*	10.19	6.52E-14	Novel
*TCONS_00004655*	−6.33	5.85E-13	Novel
*TCONS_00028787*	9.42	9.13E-13	Novel
*TCONS_00073348*	−4.55	9.13E-13	Novel
*TCONS_00076198*	8.70	9.13E-13	Novel
*TCONS_00018288*	7.69	2.32E-12	Novel
*TCONS_00043402*	7.19	4.38E-12	Novel
*TCONS_00073351*	−5.06	5.18E-12	Novel
*TCONS_00038368*	−4.15	6.09E-12	Novel

FC, the fold change of mechanical stress compared to control. FDR, *p*-value adjusted by the Benjamini–Hochberg method. FDR < 0.05 was considered statistically significant.

### Notable Involvements of lncRNAs in ceRNA Regulation Networks

**FIGURE 4:**
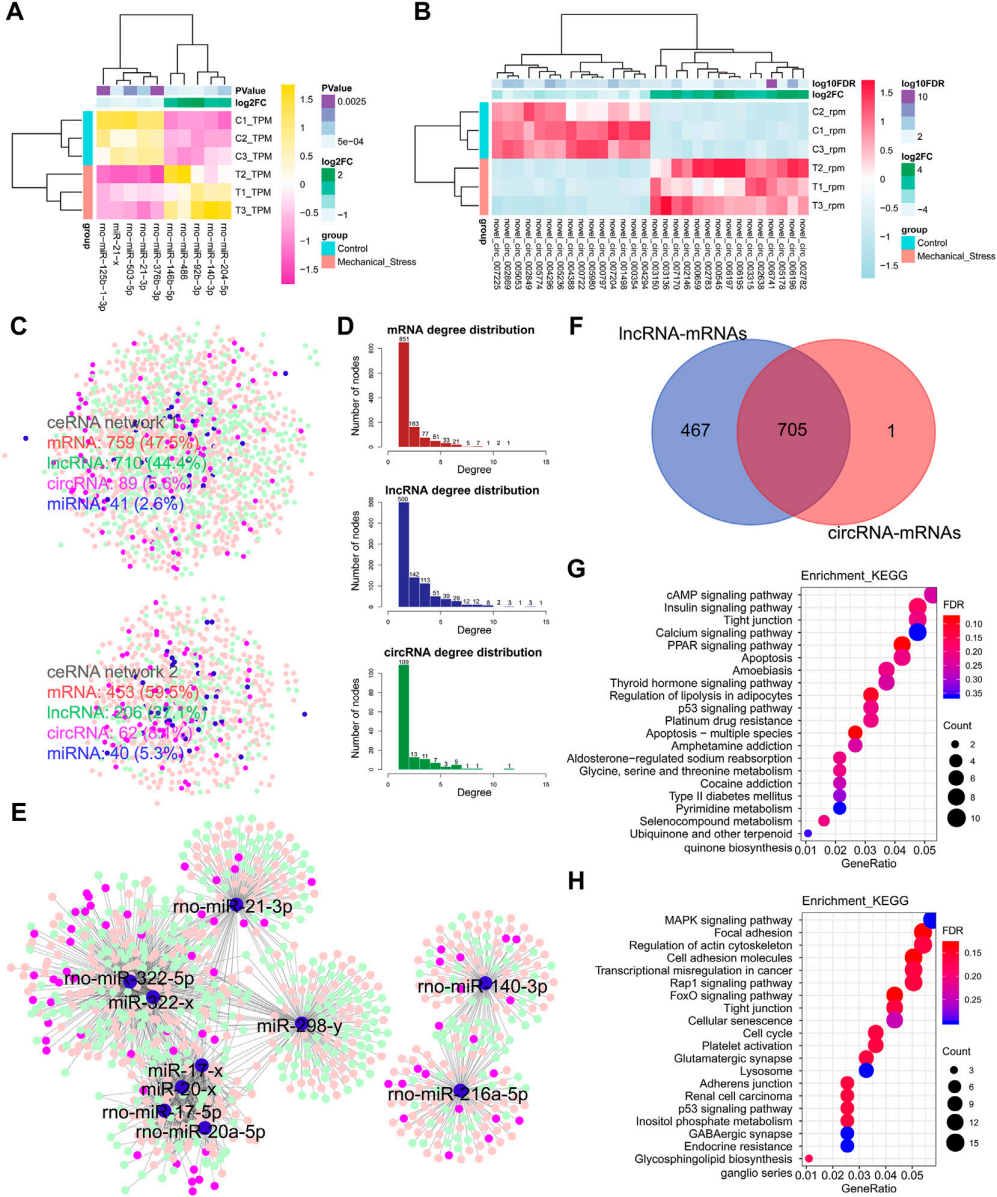
Notable involvement of lncRNAs in ceRNA regulation networks. **(A)** Top 5 up- or downregulated miRNAs (DEMIs) in the rank of *p*-value. **(B)** Top 15 up- or downregulated circRNAs (DECs) in the rank of FDR. **(C)** miRNA-centric ceRNA regulation networks constructed by DEMs (red), DEMIs (blue), DELs (green), and DECs (purple). In the ceRNA network 1, DEMs, DELs, DECs, and DEMIs account for 47.5%, 44.4%, 5.6%, and 2.6%, respectively. In the ceRNA network 2, DEMs, DELs, DECs, and DEMIs account for 59.5%, 27.1%, 8.1%, and 5.3%. **(D)** The degree distribution of DEMs, DELs, and DECs in ceRNA networks. The degree is defined as the number of nodes connected. The degree > or = 5 is defined as high connectivity in networks. The DELs with high connectivity are more than the DEMs, while the DELs with the degree lower than 5 are less than DEMs. **(E)** The essential miRNA-centric subnetworks. **(F)** The overlap of the DEMs connected to DELs or DECs. A total of 705 DEMs are defined as the commonly regulated genes by lncRNAs and circRNAs, accounting for >99.9% in the DEMs regulated by circRNAs. A total of 467 DEMs are defined as the lncRNA specifically regulated genes. **(G)** The KEGG pathway analysis of the lncRNA specifically regulated genes by the ORA method. The color and size of dot represent FDR and count of the genes assigned into the corresponding KEGG pathway, respectively. FDR, false discovery rate, *p*-value estimated by one-way ANOVA and adjusted by the Benjamini–Hochberg method. GeneRatio indicates the gene number ratio in each KEGG pathway. **(H)** The KEGG pathway analysis of the commonly regulated genes by the ORA method.

Besides the mechanisms of *cis*-, *trans-*, and *antisense* regulation, lncRNAs are also involved in ceRNA regulation networks and regulate target coding genes by the crosstalk with miRNAs and circRNAs. In this context, we also constructed the potential ceRNA regulation networks based on DEMs, DELs, DEMIs, and DECs. In Figures 4A,B, the top 5 up- or downregulated DEMIs and top 15 up- or downregulated DECs were identified as the outstanding responsive DEMIs or DECs. Two miRNA-centric ceRNA regulation networks were constructed, including networks 1 and 2. In network 1, mRNAs, lncRNAs, circRNAs, and miRNAs accounted for 47.5%, 44.4%, 5.6%, and 2.6%, respectively. A decreased proportion of lncRNAs (∼38.96%) and an increased proportion of circRNAs (∼44.64%) happened in network 2, while the numbers of miRNAs of both networks were comparable (Figure 4C). Given that the proportions of lncRNAs in both ceRNA networks were largest among ncRNAs, we speculated that lncRNAs may play a more critical role and serve as the hubs in network. Moreover, we calculated the degree of each composition, defined as the number of nodes, in ceRNA networks (Figure 4D). Indeed, the numbers of lncRNA hubs with the high degree > or = 5 were higher than both mRNAs and circRNAs. Moreover, two miRNA clusters *miR-322*/*miR-20a*/*miR-17/miR-21/miR-298* and *miR-140*/*miR-216* were identified in the subnetworks (Figure 4E). Interestingly, 705 overlapped DEMs connected to DELs or DECs were defined as the commonly regulated genes by lncRNAs and circRNAs, accounting for >99.9% of the DEMs regulated by circRNAs, while 467 DEMs were defined as the lncRNA specifically regulated genes. To know the underlying functions of lncRNAs in ceRNA networks, these two groups of genes were subjected to KEGG pathway analysis (Figures 4G,H). The lncRNA specifically regulated genes were mainly involved in metabolism pathways, such as glycine, serine, and threonine metabolism (Supplementary Figure S8) and type II diabetes mellitus, pyrimidine metabolism, and selenocompound metabolism, while both groups were involved in the pathways relevant to stress, apoptosis, and cell cycle.

## Discussion

To understand the underlying ncRNA mechanisms of mechanical stress in OA pathogenesis, we employed RNA-seq to define the ncRNA transcriptome remodeling, characterized by thousands of differentially expressed mRNAs, lncRNA, miRNAs, and circRNAs and their complicated regulation relations, which provides a full landscape of transcriptome response. Moreover, these responsive ncRNAs are potentially biomarkers and therapeutic targets that could be modified in OA therapy as well as cartilage regeneration, such as implantation of pre-modified chondrocyte based on these ncRNAs.

In our study, we first defined the differentially expressed transcripts in each composition of whole transcriptome. In spite of the fact that mRNA composition has the largest number of differentially expressed transcripts, the ratio of DELs in lncRNA composition is up to 18.9%, the largest compared to others (mRNA: 12.8%, miRNA: 5.8%, circRNA: 5.4%), suggesting that lncRNAs are more responsive to mechanical stress. We speculated more notable roles of lncRNAs in whole transcriptome compared to miRNAs or circRNAs. Due to this speculation, we focused on the lncRNA composition and the relationship with the others in data analysis. Interestingly, we found more than 3,000 lncRNAs without the known annotation, which we defined as the novel lncRNAs. Moreover, mechanical stress gives promise to the novel lncRNA expression, and the number of the novel DELs was more than 10-fold of the known DELs, which suggests that this part is more responsive to mechanical stress in the lncRNA transcriptome. Indeed, the roles of lncRNAs are highlighted in chondrocyte biology and OA pathogenesis (Tu et al., 2020; van Hoolwerff et al., 2020), such as lncRNA *Pvt1* (Li et al., 2017). In spite of the fact that increased *Pvt1* is proven to be a promoter of chondrocyte apoptosis contributing OA, we found that *Pvt1* was decreased instead in our system, suggesting that apoptosis might not be robustly induced by this mechanical stress. Indeed, the apoptosis-relevant pathways were not significantly responsive as the others reported (Kong et al., 2013; Xu et al., 2019), while a group of apoptosis-relevant DEMs were involved in this process. Except for a unique form of OA resulting from clear trauma, namely, post-traumatic osteoarthritis (POTA), chondrocyte apoptosis is a final outcome in late OA, while the effect of mechanical stress lasts throughout the lifespan, which may not be a pivotal inducing factor but an accumulated influence on chondrocyte fate. At the same time, we also found decreased proliferation and blocked cell cycle, indicating that the stimulation in our system induced cell senescence. Indeed, the senescent chondrocytes were increased under this mechanic system.

Non-random positioning of lncRNAs in genome implies a connection between lncRNA function and the genomic neighborhood (Statello et al., 2021). lncRNAs regulate neighborhood in *cis*-acting regulation (locally), a special form of which depends on the interaction between antisense lncRNAs and host genes, namely, *antisense-*regulation (Villegas and Zaphiropoulos, 2015). Besides this *cis*-acting regulation, lncRNAs also regulate distal protein-coding genes in *trans* (non-locally) (Yan et al., 2017). The knowledge of lncRNA-mediated *cis-*, *trans*-, and *antisense-*regulation is critical for our understanding of RNA-mediated gene regulation and genome complexity of mechanical response. Before that, we first need to understand the response of coding genes and then build the connection of coding genes and lncRNAs to define the functions of mechanical-responsive lncRNAs. In the functional annotation of coding genes, we found that the robust responses of signaling pathways lay on enhanced cell metabolism and immune response. Up to now, no evidence supports the involvement of lncRNA in metabolism modification in mechanical stress-induced chondrocytes, while it is widely accepted that mechanical stimulation alters cartilage and chondrocyte metabolism (Zheng et al., 2021; Bleuel et al., 2015). Our study revealed some metabolism-relevant lncRNA-coding gene pairs, such as *TCONS_00015386*-*Adcy5*. *Adcy5* encodes a member of the membrane-bound adenylyl cyclase enzymes involved in various signaling pathways, such as cGMP-PKG (Gorbe et al., 2010) and cAMP (Vatner et al., 2015) signaling pathways, suggesting that mechanics-responsive *TCONS_00015386* may also involve these pathways.

Pereira et al. (2016) showed that chondrocytes involve immune response by directly affecting T-cell proliferation and indirectly inhibiting monocyte differentiation, indicating the native functions of chondrocyte in immune regulation and also the underlying immune-relevant mechanisms of chondrocytes in OA pathogenesis. Indeed, a strong relevance of the mechanics-responsive lncRNAs to metabolism and immune pathway suggests a potential involvement of lncRNAs in the response to mechanical stress. Immune-related lncRNAs are being identified during the activation of the innate immune response and T-cell development, differentiation, and activation (Heward and Lindsay, 2014). In our study, some immune-relevant lncRNAs-coding gene pairs were identified, such as *TCONS_00029778*-*Cd80*. Given that CD80 plays a critical role in the initiation of T-cell responses and Pereira et al. (2016) built the link between T cells and chondrocytes, we speculated that lncRNA-mediated CD80 elevation in mechanical-induced chondrocytes may be involved in the immune mechanisms of OA pathogenesis. Indeed, the development of antigen-induced arthritis was significantly suppressed by inhibition of CD80 (Odobasic et al., 2008). Also, Ji et al. (2019) identified a subpopulation of chondrocytes by single-cell sequencing, namely, regulatory chondrocytes (RegCs), which highly expresses *CD74*, *CD80*, *CD86*, and *HLADPA1* and involves immune-relevant signaling pathway, such as Toll-like receptor, JAK/STAT, and chemokine signaling. This funding gives a novel layer of understanding that chondrocytes are directly involved in immune response. These lines of evidence provide new insights and potential therapeutic directions of targeting-CD80 and CD80-relevant lncRNAs in overloading-induced cartilage degeneration.

Besides the transcriptional regulations, lncRNAs regulate gene expression in ceRNA mechanisms at the post-transcriptional level (Dykes and Emanueli, 2017; He et al., 2019). Accumulating numbers of studies have demonstrated the ceRNA mechanism in chondrocyte biology and OA development (Liu et al., 2016c). In our study, the significant interaction of miRNAs, lncRNAs, and circRNA was identified as a complicated ceRNA regulatory network, in which lncRNAs showed more outstanding involvements compared to circRNAs. Indeed, some lncRNAs were proven to be critical regulators in ceRNA mechanism, such as lncRNA *ADAMTS9-AS2* (Huang et al., 2019), *DANCR* (Zhang L et al., 2018), and *FOXD2-AS1* (Cao et al., 2018). We found that lncRNAs specifically regulate amino acid (AA) metabolism pathways, such as glycine, serine, and threonine metabolism and pyrimidine metabolism, in ceRNA mechanisms. Alterations of AA metabolism, such as glutamate- and arginine-family AA as well as their related metabolites, have also been identified in OA (Li et al., 2016). At the same time, the involvements of miRNAs and circRNA are also indispensable in the ceRNA network. Among miRNA clusters we identified, *miR-20a* (Zhao and Gong, 2019), *miR-21* (Sekar, 2020), *miR-322* (Georgieva et al., 2020), and *miR-17* (Hu et al., 2018) have been validated in OA pathogenesis and *miR-298* is responsive to IL-1β in OA chondrocyte (Akhtar et al., 2010). Moreover, *miR-140* has a critical role in cartilage development and homeostasis (Araldi and Schipani, 2010) as well as OA pathogenesis (Miyaki et al., 2009) and *miR-216a-5p* regulates the proliferation and apoptosis of chondrocytes in OA by targeting the JAK-STAT signaling pathway (Zhang L et al., 2018). These lines of evidence suggest the significance of these miRNA clusters and the underlying mechanisms of mechanical stress by regulating these miRNAs.

In our previous comparison study (Zhang et al., 2021), we found the different or same effects between moderate (2,000 μstrain) and extensive (5,000 μstrain) mechanical stress on chondrocyte mitochondrial functions. We observed the effects of mechanical stress on mitochondrial functions and cell fate, or more specifically, apoptosis. We found a beneficial effect of 2,000 μstrain on cell survival, while we did not consider the impact on anabolic and catabolic metabolisms of chondrocytes. The previous findings seem to be contradicted to the present study. Actually, cellular senescence is defined as an apoptosis-resistant state with increased senescence-associated secretory phenotype (SASP). Of note, *Mmp13* is a well-characterized component of SASP. In the present study, we found increased senescent chondrocytes under 2,000 μstrain, suggesting more apoptosis-resistant cells as we found in the previous study. Moreover, we found that the co-location of mitochondria and lysosomes was increased under this stimulation, which suggests the potential enhanced mitophagy. This is coherent with our previous finding that 2,000 μstrain is able to promote mitochondrial dynamic and mitophagy. Taking these two studies together, we concluded that 2,000 μstrain mechanical stress can induce OA phenotypes partially in an *in vitro* model, characterized by increased MMPs and accumulated cellular senescence. Chondrocyte senescence appears to be a compromise for survival in mechanical stress. The distinct effects of this stress may lie on the different responses of subpopulations among the primary chondrocytes, while our findings are based on the bulk effects of overall chondrocytes.

The limitations of this study should be discussed carefully. Firstly, we employed IL-1β-treated rat/rodent OA-like chondrocytes but not human OA chondrocytes isolated from patients. Also, our conclusion is based on the 5 ng/ml IL-1β treatment because we did not employ the multiple concentrations of IL-1β. Secondly, we employed only one mechanical model in this study, while chondrocytes are assumed to be subjected to multiple kinds of mechanical stimulation *in vivo*. Thirdly, we did not include the normal chondrocytes treated with mechanical stimulation but not IL-1β. As a result, we did not conclude the effects of mechanical stimulation in normal chondrocytes. Whether the effects of mechanical stimulation on OA-like chondrocyte transcriptome is IL-1β-dependent or not is still inconclusive. Finally, the low homology between human and rodent lncRNAs would hammer the significance of this study, while the functions of some lncRNAs are assumed to be resistant to evolutionary constraints at both RNA sequence and structural levels due to their critical roles (Karner et al., 2020).

## Conclusion

In conclusion, mechanical stress reprograms whole transcriptome of IL-1β-induced OA-like chondrocytes and drives disruptions of signaling pathways. The mechanics-responsive lncRNAs may be critical in mechanical stress-induced metabolism and immune modifications. Further research into the functions of these genes and their interaction with lncRNAs may lead to a better understanding of their biogenesis and the mechanism underlying OA, and thus, they may eventually be used as diagnostic biomarkers and therapeutic agents for OA.

## Data Availability

The datasets presented in this study can be found in online repositories. The names of the repository/repositories and accession number(s) can be found in the article/Supplementary Material.

## Abbreviations

AA, amino acid; Aqp8, Aquaporin 8; Acan, aggrecan; CPC, Coding Potential Calculator; CNCI, Coding-Non-Coding Index; CeRNA, endogenous RNA; CircRNAs, circular RNAs; Col2a1, collagen II alpha1; Cstf2, Cleavage stimulation factor subunit 2; Cyp1a1, Cytochrome P450 family subfamily A member 1; DECs, differentially expressed circRNAs; DELs, differentially expressed lncRNAs; DEMs, differentially expressed mRNAs; DEMIs, differentially expressed miRNAs; ECM, extracellular matrix; GO, gene ontology; GSEA, gene set enrichment analysis; LncRNAs, long-noncoding RNAs; KEGG, Kyoto Encyclopedia of Genes and Genomes; Nisch, Nischarin; MiRNAs, microRNAs; MMPs, metalloproteinases; NcRNAs, noncoding RNAs; OA, osteoarthritis; PCA, principal component analysis; RT-PCR, real-time polymerase chain reaction; RT, reverse transcription; Srebf1, Sterol regulatory element-binding transcription factor 1; Trp53inp1, Tumor protein P53 inducible nuclear protein 1; Slc30a2, Solute carrier family 30 member; Wbp4, WW domain binding protein 4; Zc3h12a, Zinc finger CCCH-type containing 12A.
