# Supplementary figures and images for: Whole Transcriptome Mapping Identifies an Immune- and Metabolism-Related Non-coding RNA Landscape Remodeled by Mechanical Stress in IL-1β-Induced Rat OA-like Chondrocytes
Source: Front Genet. 2022 Mar 3;13:821508. doi: 10.3389/fgene.2022.821508 (PMC8927047; doi:10.3389/fgene.2022.821508)

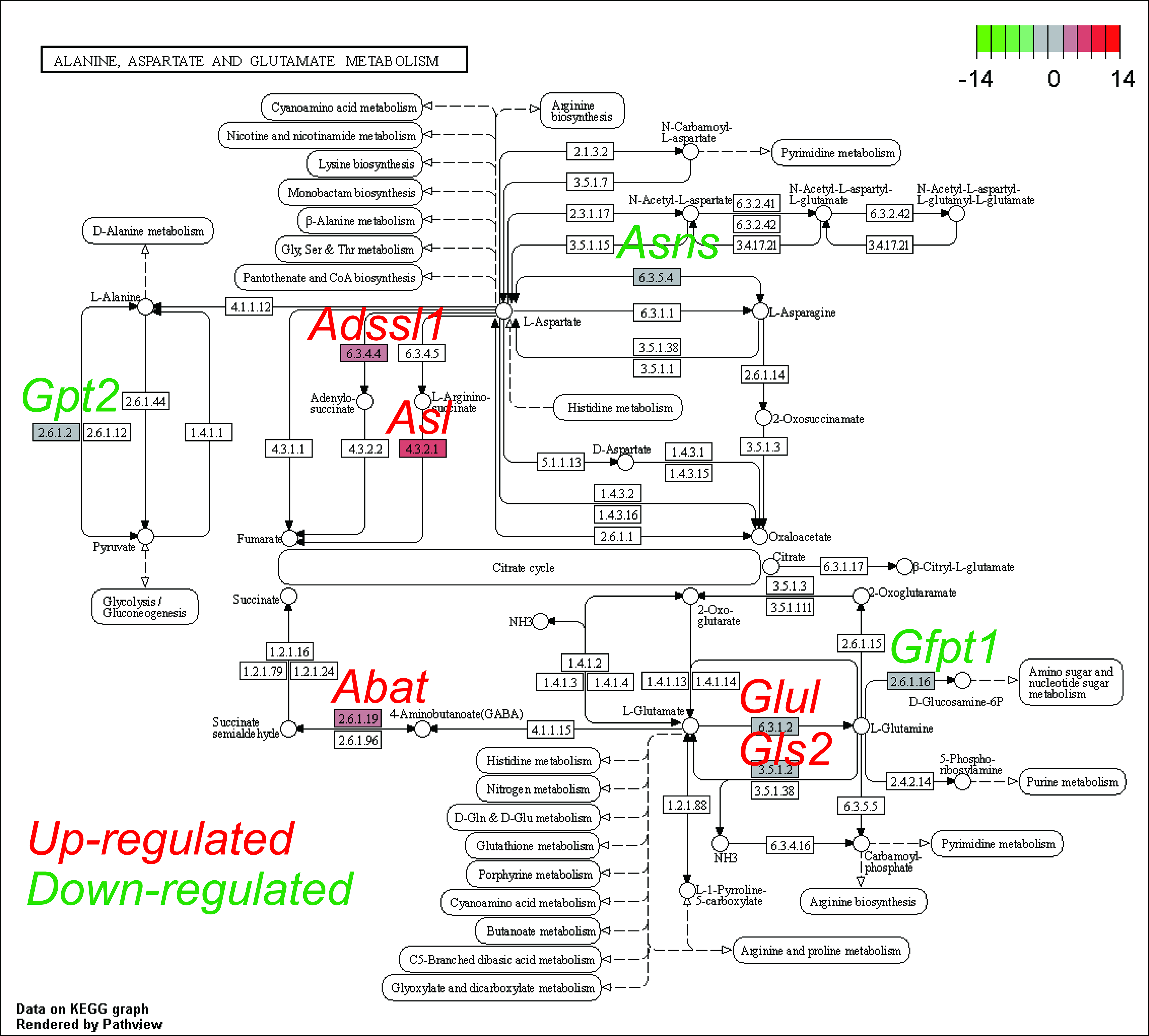

Supplement: Supplementary file 2 [file Image6.TIF]

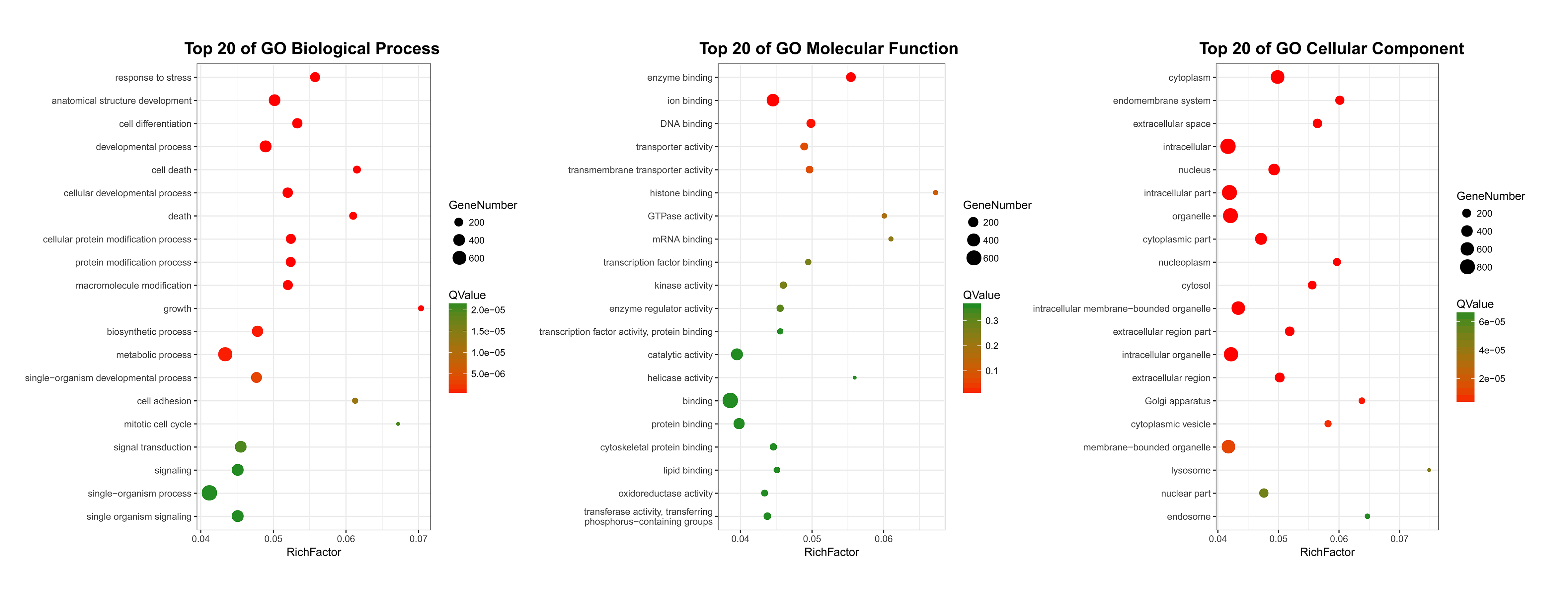

Supplement: Supplementary file 4 [file Image3.TIF]

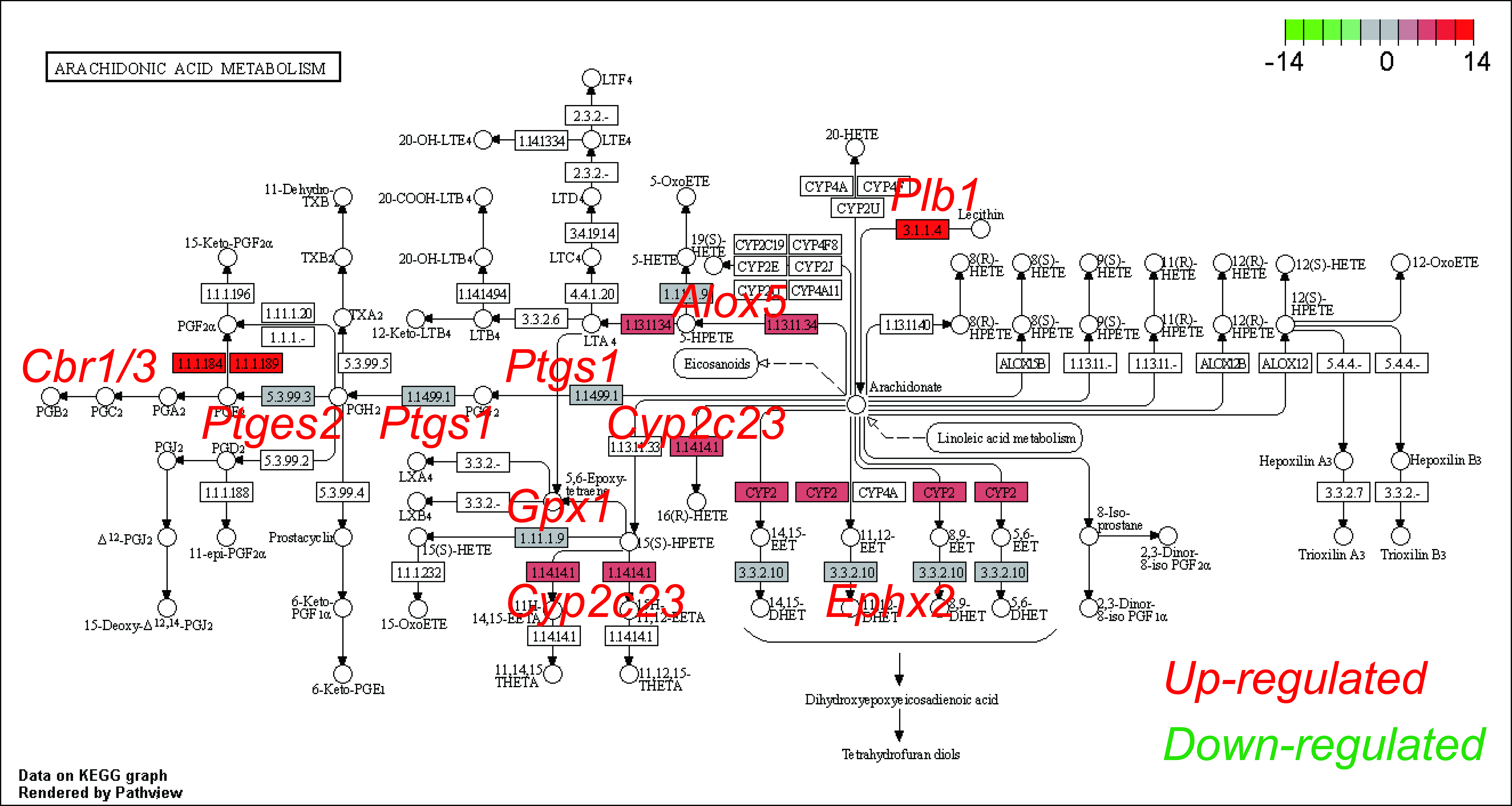

Supplement: Supplementary file 5 [file Image4.TIF]

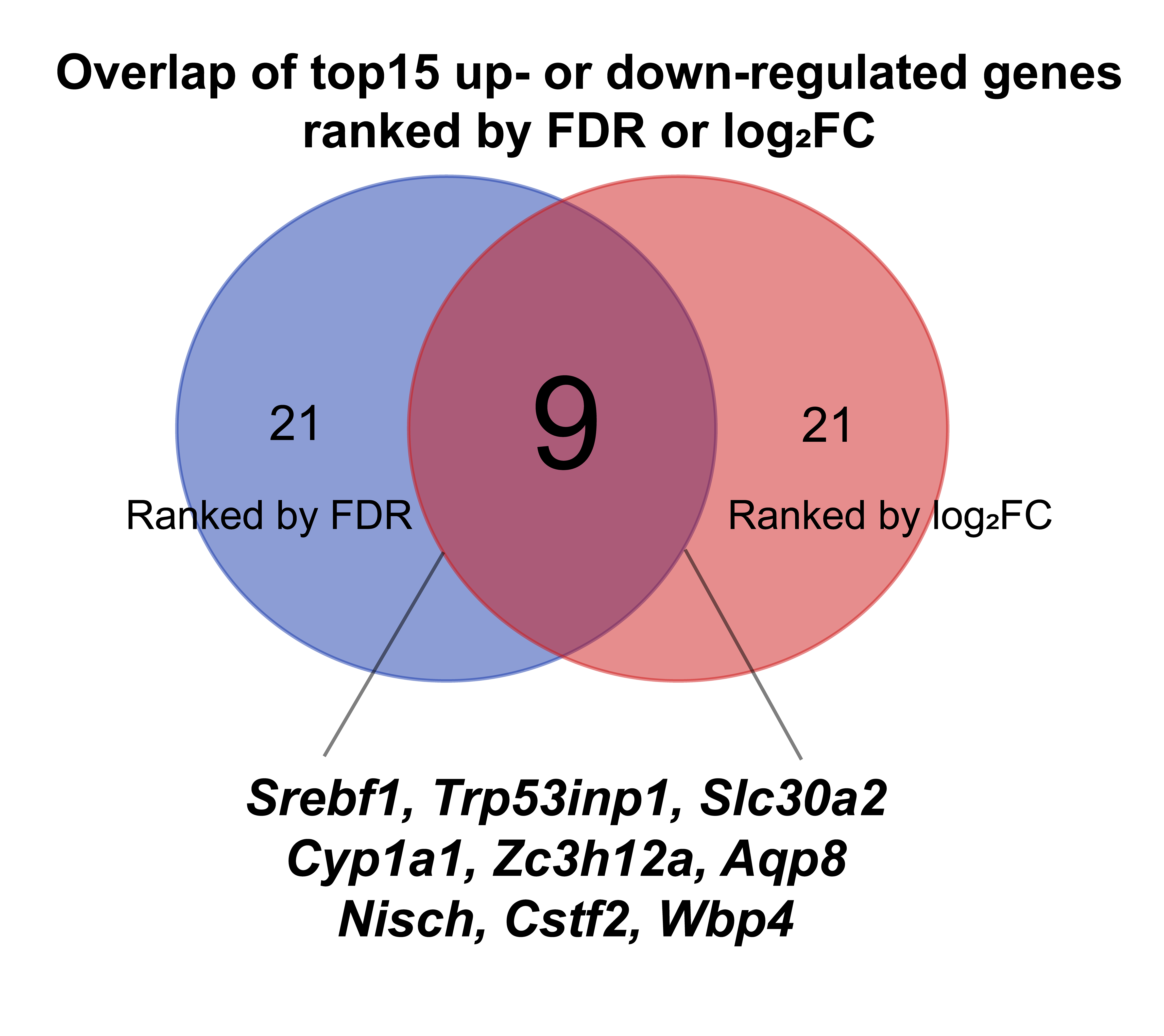

Supplement: Supplementary file 6 [file Image2.TIF]

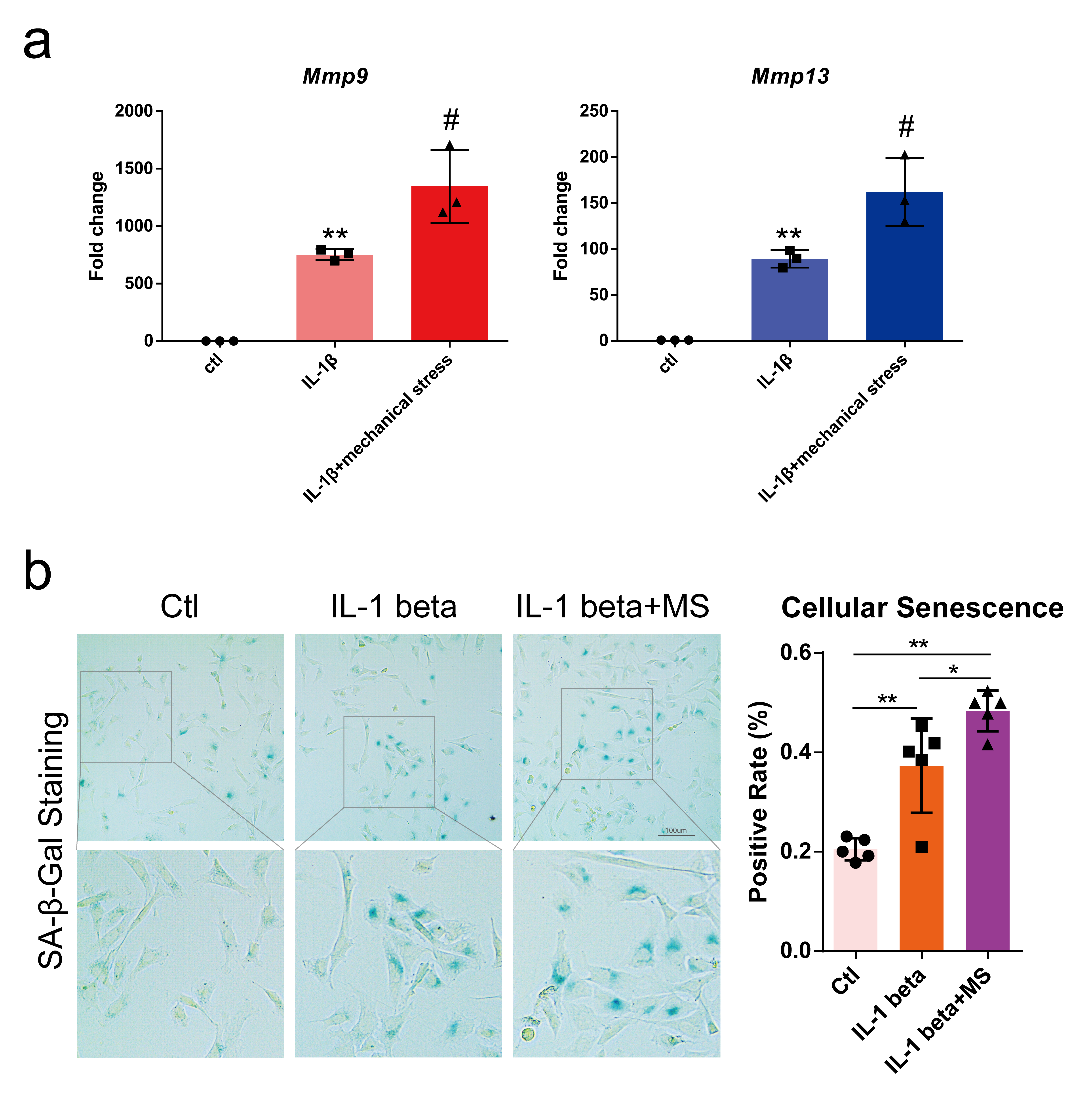

Supplement: Supplementary file 7 [file Image1.TIF]

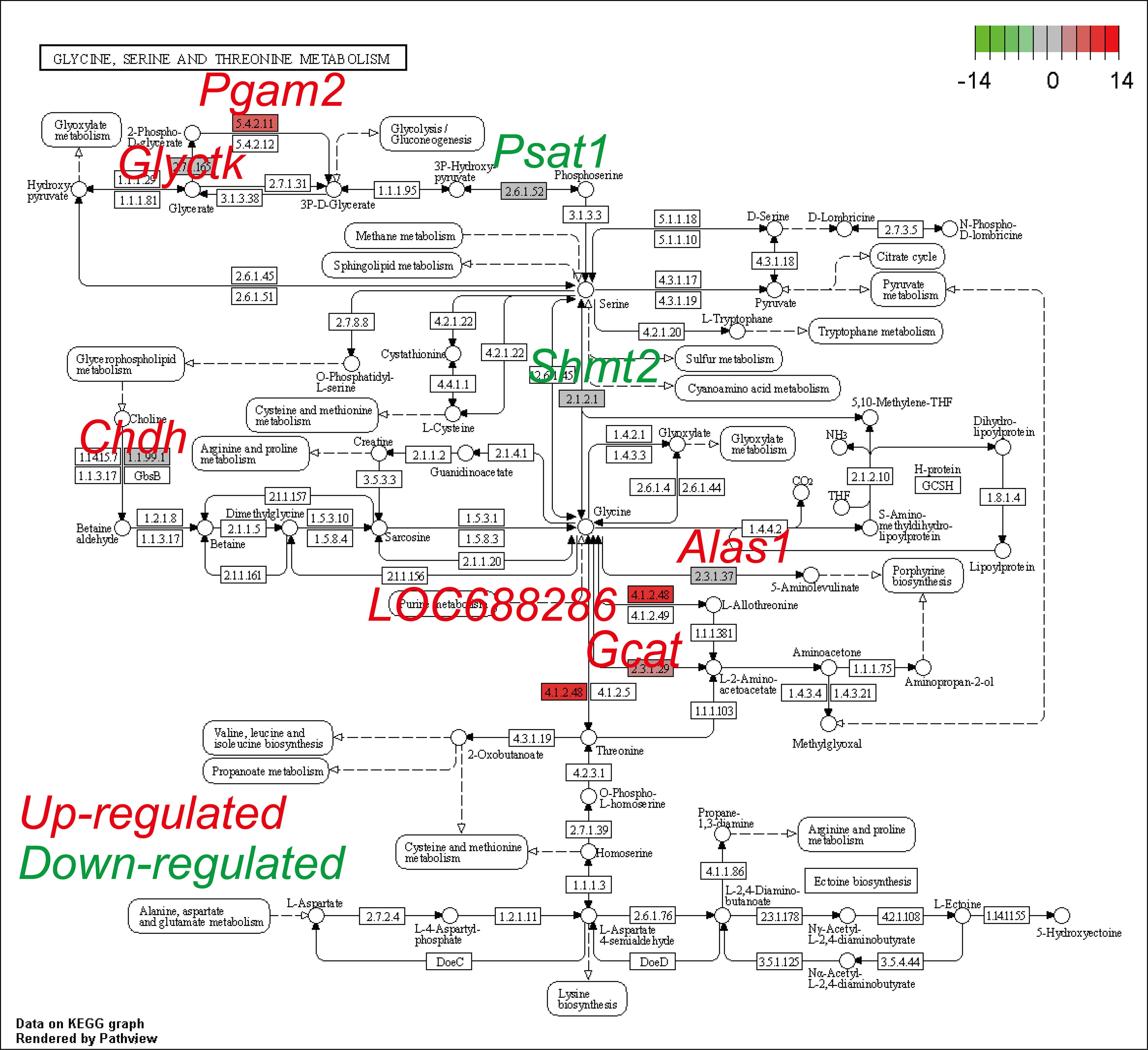

Supplement: Supplementary file 8 [file Image7.TIF]

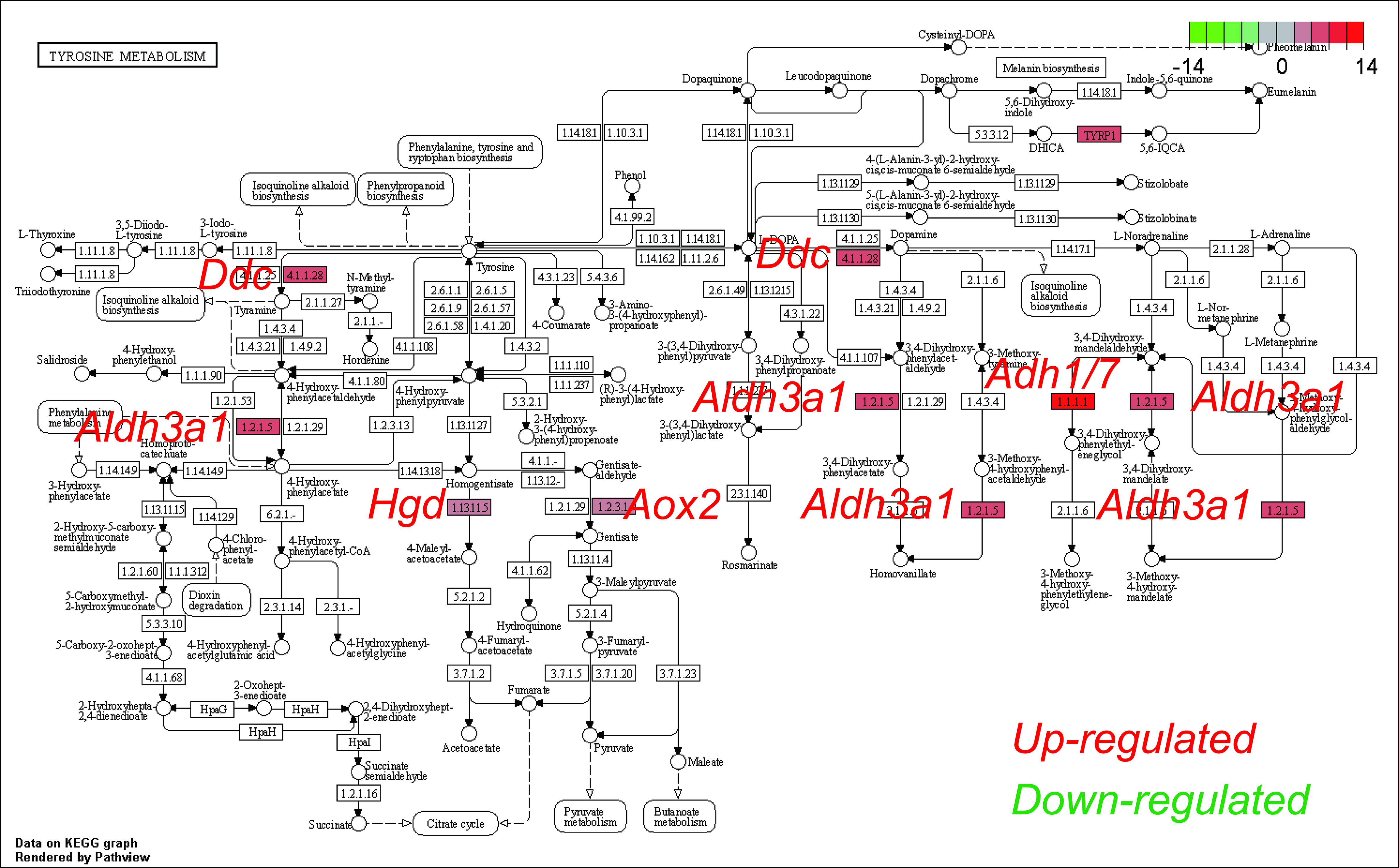

Supplement: Supplementary file 9 [file Image5.TIF]
