# Supplementary material for: Whole Transcriptome Mapping Identifies an Immune- and Metabolism-Related Non-coding RNA Landscape Remodeled by Mechanical Stress in IL-1β-Induced Rat OA-like Chondrocytes
Source: Front Genet. 2022 Mar 3;13:821508. doi: 10.3389/fgene.2022.821508 (PMC8927047; doi:10.3389/fgene.2022.821508)
Supplement: Supplementary file 3 [file Table1.DOCX]

**Supplemental Table 1. Primer sequences for real-time RT-PCR.**

| **Gene Symbol** | **Primer Forward** | **Primer Reverse** |
| --- | --- | --- |
| ***Mmp13*** | 5'-CCCAGCCCTATCCCTTGATG-3' | 5'-TCGGGATGGATGCTCGTATG-3' |
| ***Mmp9*** | 5'-TCGGATGGTTATCGCTGGTG-3' | 5'-AAGACGCACATCTCTCCTGC-3' |
| ***Gapdh*** | 5'-CCTTCTCTTGTGACAAAGTGGACAT-3' | 5'-GCCCACTTCTCAGGCACATC-3' |
| ***Rock2*** | 5'-GCTTTTGCTAACAGTCCGTGG-3' | 5'-GCCCACTTCTCAGGCACATC-3' |
| ***Aldh3a1*** | 5'-CCCTCTTTCAGCTCTTGCTCA-3' | 5'-GAGGTCCATTCGTTCTTGCC-3' |
| ***Vamp8*** | 5'-CCTGGACCATCTCCGAAACAA-3' | 5'-GGTGCCCGTAGCAAAGAGTATG-3' |
| ***Slc30a1*** | 5'-CAGGAGGAGACCAACACGCT-3' | 5'-CAACAACGATCACAGAGCCCA-3' |
| ***Cyp1a1*** | 5'-AGCACTACAGGACATTTGAGAAG-3' | 5'-AGGCTCCAAGAGATAGCAGTT-3' |
| ***Npr1*** | 5'-TGGCACTTGCACTACTGGAT-3' | 5'-CATTTCCACATCCCCTCGGA-3' |
| ***Srebf1*** | 5'-TGCGAAGTGCTCACAAAAGC-3' | 5'-GGAGGGGTCAGCGTTTCTAC-3' |
| ***Pvt1*** | 5'-CCCTCCCGCCTGATTTTCTT-3' | 5'-TCTAGGCAGGCCAGGTGTAT-3' |
| ***AC127756.1*** | 5'-TGAAAAGTGCCAGTGAGGTGTG-3' | 5'-GCTGTGGTGTAGATGTATTTGTTAGG-3' |
| ***TCONS_00028770*** | 5'-TGACCCTGAAGAAAACCAACG-3' | 5'-GACCAAGACAGCCAAAATCCC-3' |
| ***TCONS_00062413*** | 5'-CCATTTTCCTTGGTCTTTTGATA-3' | 5'-ACAGGCTTTCTTGGAGCTCTC-3' |
| ***Cd80*** | 5'-TGCTCACGTGTCACAGAACT-3' | 5'-TGAAGCAGCGTCGGAATTTG-3' |
| ***TCONS_00029778*** | 5'-GGTTCAGGGTGAGTTCGGTT-3' | 5'-CAGATGTGCCCTGAAAAGCC-3' |

The primers were designed using Primer-blast (<https://www.ncbi.nlm.nih.gov/tools/primer-blast>) for PCR.
